# Ultra-sensitive, tumor-informed ctDNA profiling in pembrolizumab-treated esophagogastric cancer patients predicts clinical responses

**DOI:** 10.21203/rs.3.rs-5349536/v1

**Published:** 2024-12-18

**Authors:** Andrew B. Nixon, Fábio C. P. Navarro, Katherine I. Zhou, Charles Abbott, Lee McDaniel, Lauren Howard, J Christopher Brady, Yingmiao Liu, Jingquan Jia, Donna Niedzwiecki, John Strickler, Sean M. Boyle, Richard O. Chen, Hope Uronis

**Affiliations:** Duke University Medical Center; Personalis (United States); Duke University Medical Center; Personalis (United States); Personalis (United States); Duke University; Duke University Medical Center; Duke University Medical Center; University of North Carolina at Chapel Hill; Duke University; Duke University Medical Center; Personalis (United States); Personalis (United States); Duke University Medical Center

**Keywords:** ctDNA, personalized assay, ultra-sensitive detection, gastroesophageal cancer, pembrolizumab

## Abstract

To explore whether ultra-sensitive circulating tumor DNA (ctDNA) profiling enables early prediction of treatment response and early detection of disease progression, we applied NeXT Personal, an ultra-sensitive bespoke tumor-informed liquid biopsy platform, to profile tumor samples from the KeyLargo study, a phase II trial in which metastatic esophagogastric cancer (mEGC) patients received capecitabine, oxaliplatin, and pembrolizumab. All 25 patients evaluated were ctDNA-positive at baseline. Minimal residual disease (MRD) events varied from 406,067 down to 1.5 parts per million (PPM) of ctDNA with a median limit of detection of 2.03 PPM. ctDNA dynamics were highly correlated with changes in tumor size (ρ = 0.59, *p* = 7.3×10^−9^). Lack of early molecular response (lack of ctDNA decrease) was associated with worse overall survival (OS) (HR 6.6, 95% CI 1.8-24.1, *p* = 0.005) and progression-free survival (PFS) (HR 15.4, 95% CI 2.7-87.0, *p* = 0.002). Lack of molecular clearance of ctDNA was associated with worse OS (HR 6.9, 95% CI 1.5-30.8, p = 0.012) and PFS (HR 19.2, 95% CI 2.4-152.8, p = 0.005). Molecular progression (ctDNA increase) preceded imaging-derived progression by a median lead time of 65 days. These results suggest that ultra-sensitive liquid biopsy approaches could improve treatment decision-making for mEGC patients receiving chemotherapy and immunotherapy.

## Introduction

Esophagogastric cancer (EGC) is the fourth most common cancer worldwide^1^. Most EGC cases are diagnosed at advanced stages, presenting a great challenge in clinical oncology due to their aggressive nature and limited treatment options^2^. Currently, first-line treatment for HER2-negative metastatic EGC consists of chemotherapy with or without immunotherapy depending on tumor PD-L1 expression^3,4^. While the addition of immunotherapy has revolutionized the landscape of EGC treatment, identifying patients who will respond favorably to these therapies remains an ongoing challenge. The absence of reliable biomarkers for predicting immunotherapy response hampers the precision of treatment strategies, leading to variable outcomes among EGC patients.

In recent years, the emergence of liquid biopsy has sparked excitement as a potential solution to this clinical gap. Liquid biopsy offers a minimally invasive approach to monitor tumor dynamics and treatment response, including the analysis of circulating tumor DNA (ctDNA) in the peripheral blood^5–7^. Unlike tissue biopsies, which are challenging to obtain serially throughout therapy, liquid biopsies can be easily collected and longitudinally profiled, allowing for more informed clinical decisions^8^.

Some studies have begun to examine the role of ctDNA as a biomarker in gastric cancer, exploring its potential in screening, disease monitoring, and risk stratification^9^. In early stages of gastric cancer, ctDNA levels appear to be proportional to the stage of cancer and the size of the tumor^10^. In addition, ctDNA detection after curative intent surgery in early stage EGC has been shown to risk stratify patients, with post-surgery ctDNA positivity being associated with shorter recurrence-free survival^11^. Despite the broad potential applications of ctDNA, less is known about the prognostic and predictive value of ctDNA and ctDNA dynamics in the context of advanced EGC.

A key challenge in enabling sensitive ctDNA testing is detecting and characterizing small fractions of tumor-derived ctDNA in large populations of background cell-free DNA (cfDNA). Tumor-informed liquid biopsy approaches involve initially profiling the somatic mutations of the patient’s tumor to inform the creation of a bespoke assay that specifically detects ctDNA harboring those mutations from the patient’s blood sample, which generally allows for more sensitive minimal residual disease (MRD) tracking than tumor-agnostic liquid biopsy approaches^12^. Many studies to date have relied on first-generation tumor-informed approaches, which typically employ exome sequencing of the patient’s tumor tissue to create a bespoke assay that can detect between 16 to 50 somatic mutations in the blood, resulting in a limit detection of approximately 100 PPM of ctDNA^13,14^. While these studies demonstrate the monitoring benefits of these tests, they have also shown some of the sensitivity limitations of these tests across different cancer indications, including potential false negatives^15^.

The KeyLargo study is a phase II single-arm trial in which 36 patients with HER2-negative metastatic EGC received capecitabine, oxaliplatin, and pembrolizumab as first-line treatment^16^. A total of 25 patients (69%) achieved complete response (CR) or partial response (PR), indicating significant clinical activity of this drug regimen. While the clinical results of the study were promising, biomarkers are needed to 1) distinguish potential responders and non-responders; and 2) enable patient risk stratification, as multiple patients who initially responded eventually developed resistance and progressed.

In this study, we applied a new tumor-informed liquid biopsy approach to assess treatment response among patients with advanced EGC enrolled in the KeyLargo trial. This new approach leverages whole-genome sequencing of each patient’s tumor to create a bespoke assay comprised of up to 1,800 somatic variants for ctDNA detection, enabling ultra-sensitive MRD detection down to 1 to 3 PPM of ctDNA^12^. Using this highly sensitive MRD assay, we examined whether ctDNA clearance and ctDNA dynamics could be used to monitor and predict disease status and outcomes in advanced EGC patients receiving a combination of chemotherapy and immunotherapy and examined the value of ctDNA monitoring as a complementary approach to imaging-based measures of response and progression.

## Results

### Bespoke ctDNA assay demonstrates ultra-sensitive detection

**Figure 1:**
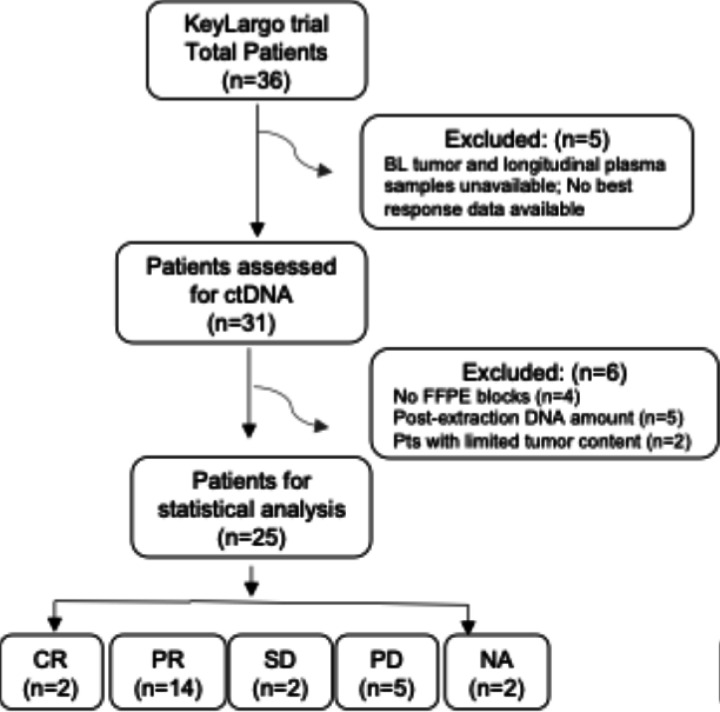
CONSORT diagram.

For the 36 advanced EGC patients enrolled in the KeyLargo trial, ctDNA analysis was performed in 25 patients. Among these 25 patients, 2 had complete response (CR), 14 had partial response (PR), 2 had stable disease (SD), and 5 had progressive disease (PD) as their best overall response. Response data was missing for two patients, specified as not available (NA) (Fig. 1). Patient characteristics for the entire study population and the biomarker cohort are shown in Supplement Table 1.

**Figure 2:**
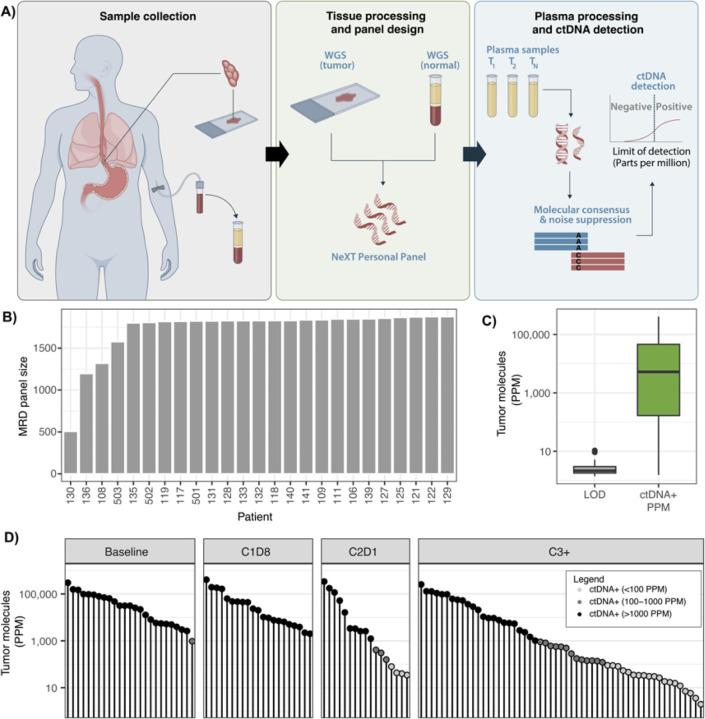
Ultra-sensitive ctDNA personalized panel design. A) Graphical depiction of the study strategy. B) Number of somatic variants included in bespoke panels for each patient. C) Distribution of limits of detection (LOD) and of PPM measures (log scale). D) Lollipop plot of all detected PPM measures. High (PPM≥1,000), medium (100≤PPM<1,000), and low (PPM<100) ctDNA levels are respectively depicted in black, dark gray, and light gray.

**Figure 3:**
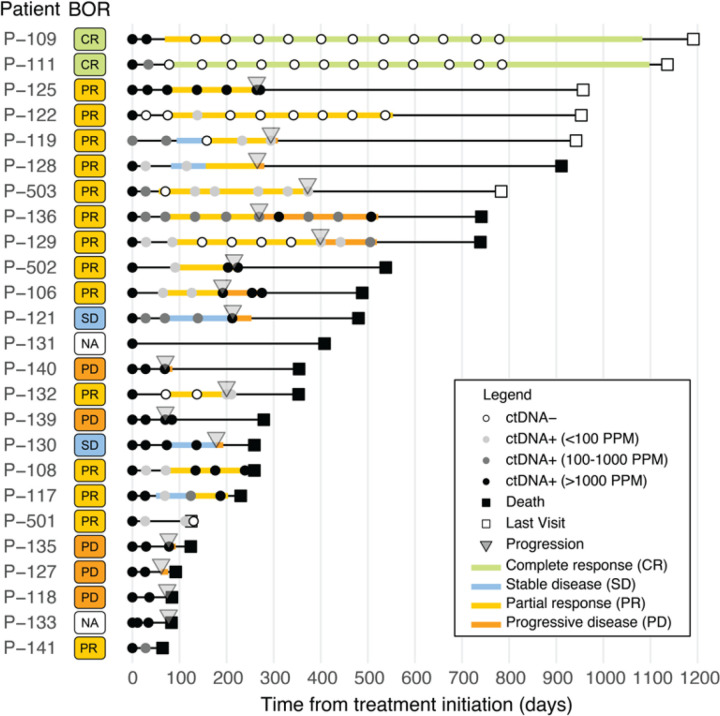
Cohort clinical and ctDNA overview. Swimmer plot of 25 patients depicting best overall response (BOR), PFS and OS events, imaging response at each imaging evaluation, and molecular response and plasma ctDNA assessment.

Bespoke liquid-biopsy panels were developed for each patient, containing probes for up to ~ 1,800 minimal residual disease (MRD) targets selected from somatic variants detected from paired tumor-normal tissue whole-genome sequencing (Fig. 2A). For the 25 evaluable patients, the DNA extracted from FFPE exhibited good quality [DNA integrity number (DIN) and fragment length] (Supplement Fig. 2 and Supplement Table 2). The resulting individual bespoke panels used to detect MRD across each patient ranged from 506 to 1,875 somatic variants. The median MRD panel size was 1,827 variants (Fig. 2B). These panels yielded a median limit of detection (LOD) of 2.03 parts per million (PPM), with quartiles spanning 1.66 to 2.74 PPM (Fig. 2C). All patients demonstrated ctDNA positivity at one or more plasma time points, and most patients exhibited ctDNA positivity across the majority of their longitudinal plasma samples (119/159) (Fig. 2D). Across all ctDNA-positive (ctDNA+) time points, we observed a broad range of ctDNA levels – from 406,067 PPM down to very low levels at 1.5 PPM (equivalent to ~ 40% and 0.00015% circulating tumor fraction, respectively). 20% (24/119) of ctDNA-positive time points displayed ultra-low ctDNA levels below 0.01% (100 PPM) in plasma. The majority of low PPM samples were measured on or after C2D1 (Fig. 2D). We noted a 100% ctDNA detection rate at baseline (C1D1) across all enrolled patients (Fig. 3).

### Early ctDNA dynamics correlate with RECIST best overall response

**Figure 4:**
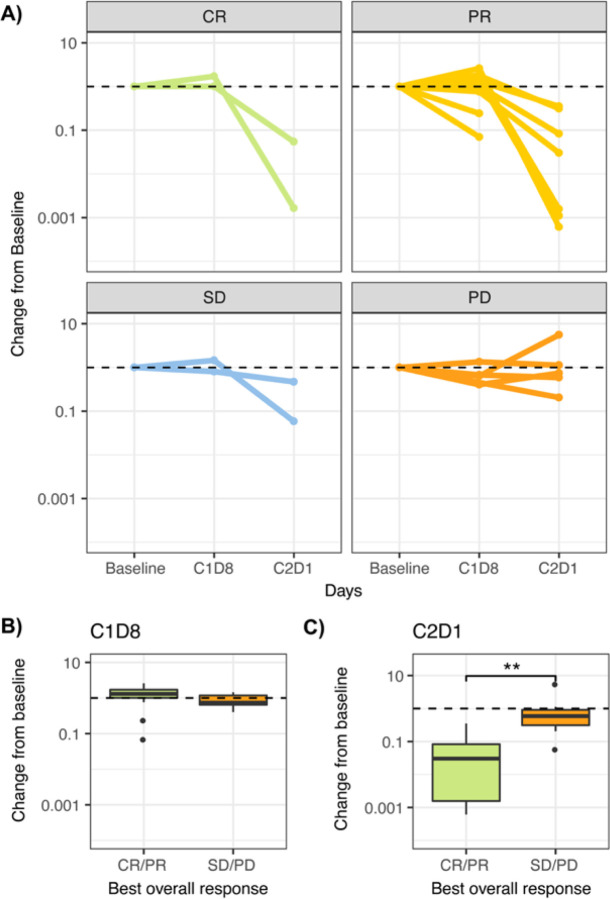
Early ctDNA dynamics differ by RECIST response. A) Spider plot showing change in ctDNA measurement from baseline over time for patients categorized by best overall response [CR: Complete response (n=2); PR: Partial response (n=14), SD: Stable disease (n=2), PD: Progressive disease (n=5)]. B) Distribution of ctDNA change from baseline to 8 days after treatment initiation (C1D8). C) Distribution of ctDNA change from baseline to approximately 28 days after treatment initiation (C2D1).

We next examined the correlation between RECIST-based best overall response and early changes in ctDNA levels from baseline. The extensive longitudinal blood sampling in KeyLargo allowed for granular analysis of ctDNA dynamics, including at one week after pembrolizumab monotherapy (C1D8) and after 28 days (C2D1) of treatment. Longitudinal changes in ctDNA levels at baseline, C1D8, and C2D1 were examined for patients in the four different response groups (CR, PR, SD, and PD) based on RECIST (Fig. 4A).

All patients were ctDNA positive until C2D1, even patients who later achieved PR/CR. Many patients exhibited an initial slight elevation in ctDNA levels at C1D8, one week after pembrolizumab monotherapy (Fig. 4B). Patients who responded to therapy (best overall response CR/PR) tended to have a larger increase in ctDNA levels at C1D8 compared to the SD/PD group (mean C1D8/baseline ratio 1.26 versus 0.88, respectively), although this difference was not statistically significant.

Extending our investigation to the C2D1 time point, a decrease in ctDNA from baseline to C2D1 was associated with improved response to therapy. For patients who were categorized as SD/PD, the median C2D1/baseline ctDNA ratio was 0.59. These patients did not show a statistically significant difference in ctDNA levels between C2D1 and baseline (One-sided Wilcoxon exact rank sum test, p = 0.355). In contrast, patients falling within the CR/PR categories displayed a significantly greater decrease in ctDNA levels, with a median C2D1/baseline ctDNA ratio of 0.015 (Fig. 4C, Wilcoxon exact rank sum test, *p* = 0.002). As expected, the difference in ctDNA level between C2D1 and baseline was statistically significant (One-sided Wilcoxon exact rank sum test, p = 0.00005). These findings demonstrate that close to 30 days after initiation of immunotherapy (C2D1), CR/PR patients experience a substantial reduction in ctDNA levels within the plasma, while ctDNA levels do not significantly change in SD/PD patients.

### ctDNA-based molecular progression often precedes imaging

**Figure 5:**
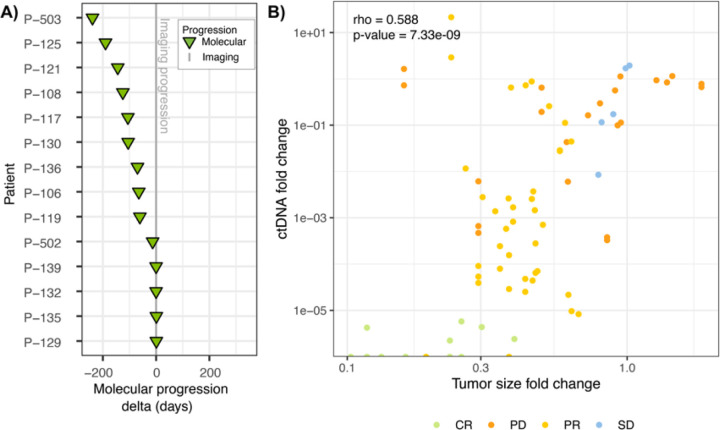
ctDNA monitoring allows for early detection of progression. A) The difference in days between molecular progression and imaging progression. Negative values indicate earlier ctDNA progression. B) Correlation between ctDNA fold change and the fold change of the sum of the longest diameters for target lesions (RECIST 1.1).

The concept of “lead time” refers to the time gained in detecting disease progression using ultra-sensitive ctDNA analyses compared to imaging methods. In this study, patients underwent frequent imaging assessments every three cycles of therapy, beginning at C4D1. Using the imaging data as a reference, the ultra-sensitive ctDNA analysis provided a median lead time advantage of 65 days (mean 55.4 days), indicating that molecular progression was detected approximately 2 months earlier than imaging-derived progression (Fig. 5A). Importantly, all patients but one (patient 140) displayed progression by ctDNA assessment prior to or at the time of progression by imaging. This patient sustained remarkably high levels of ctDNA (tumor fraction ~ 25–40%) throughout the period of measurement, and the ctDNA level measurements were the highest across the entire study.

### Longitudinal ctDNA changes correlate with RECIST tumor size

**Figure 6:**
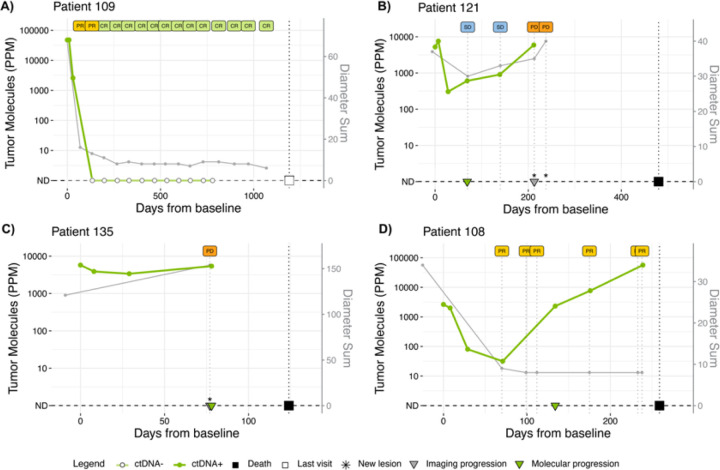
ctDNA and tumor size dynamics in select patients. Longitudinal ctDNA levels and tumor size (expressed as sum of diameters according to RECIST) in four patients whose best overall response by RECIST was: A) CR, B) SD, C) PD, or D) PR.

Our analysis also revealed a strong correlation between change in ctDNA levels and change in overall tumor burden, measured as the sum of diameters of target lesions according to RECIST criteria (Fig. 5B, Spearman’s rank correlation coefficient, ρ = 0.58, *p* = 7.3×10^− 9^). Overall, as tumor burden decreased, plasma ctDNA levels also decreased. The example case studies in Fig. 6 reflect this strong overall correlation of ctDNA levels with RECIST classifications. Patient 109 exhibited a CR and had a corresponding rapid molecular clearance of ctDNA (Fig. 6A). Patient 121 had SD followed by PD. In this case, ctDNA changes closely mirror the RECIST-based tumor size changes (Fig. 6B). In the case of patient 135, the patient was shown to be progressing both by imaging and ctDNA. However, ctDNA provided additional detail, showing that there was an initial molecular response to therapy before progression (Fig. 6C).

While most patient cases demonstrated strong correlation between ctDNA and imaging results, we also noted exceptions. For example, Patient 108 was classified as a partial responder, but the increase in ctDNA suggested molecular progression. Further review of this patient’s medical history revealed evidence of leptomeningeal metastasis on brain MRI (Fig. 6D).

### Longitudinal ctDNA changes and clearance correlate with patient survival

**Figure 7:**
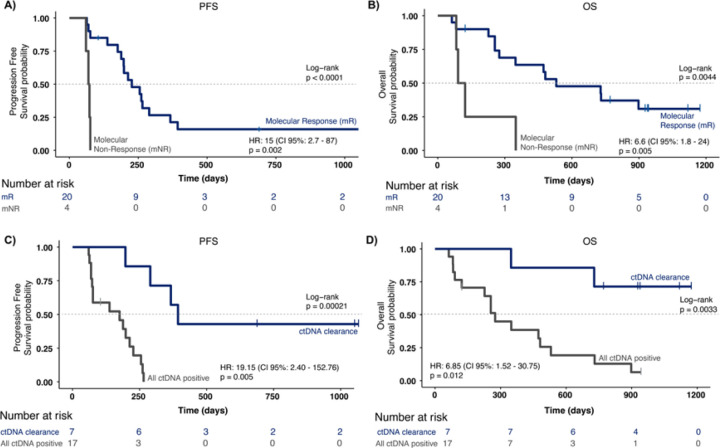
ctDNA response and clearance as prognostic biomarkers. Kaplan-Meier and univariable Cox regression analysis of A) PFS and B) OS for patients split by molecular response (mR: ctDNA ratio > 0.5 between baseline and first-time point post 10 days) and molecular non-response (mNR); Kaplan-Meier and univariable Cox regression analysis of C) PFS and D) OS for patients split by ctDNA clearance between baseline and C7D1.

To assess the relationship between longitudinal ctDNA dynamics and prognosis, we next evaluated the association of survival with the change in ctDNA levels from baseline to C2D1, or C4D1 when the C2D1 time point was not available. We divided the cohort into two groups: patients whose ctDNA levels decreased by more than 50% relative to baseline (indicating molecular response, or mR) and patients whose ctDNA levels increased, stayed the same, or decreased by less than 50% (molecular non-response, or mNR). Patients with molecular non-response had significantly inferior PFS (Fig. 7A) and OS (Fig. 7B) compared to patients with molecular response, based on log-rank test (*p* < 0.0001 for PFS; *p* = 0.0044 for OS) and Cox regression analysis (PFS: HR 15.4, 95% CI 2.72-87.00, *p* = 0.002; OS: HR 6.56, 95% CI 1.78-24.14, *p* = 0.005). Notably, of the 4 patients with molecular non-response, no patients (0/4) had a response by RECIST, and all patients (4/4) had a PFS event (RECIST progression or death) within 6 months of treatment initiation.

To explore the potential of ctDNA as a biomarker for survival, we further examined the association of survival with ctDNA clearance at any time point between C1D8 and C7D1. Patients were divided into two groups: patients who had cleared ctDNA at any of these time points (7 patients) versus those who remained ctDNA positive throughout these time points (17 patients). Kaplan-Meier survival analysis showed a significant separation between these two groups for both PFS (Fig. 7C, p = 0.0002) and OS (Fig. 7D, p = 0.0033). Failure to clear ctDNA was associated with inferior PFS (HR 19.15, 95% CI 2.40-152.76, p = 0.0053) and OS (HR 6.85, 95% CI 1.52-30.75, p = 0.012).

### Impact of ultra-sensitive ctDNA assay

We next looked at the impact of using an ultra-sensitive MRD assay like the one used in this study compared to a less sensitive MRD assay. As previously noted, many commercially available MRD tests have a limit of detection of ~ 100 PPM^14^, which suggests that ctDNA levels below that would often be undetected. In our study, ctDNA detections were as low as 1.5 PPM, and ~ 20% of all ctDNA positive detections were below 100 PPM.

To simulate what the ctDNA results would have been with a 100 PPM limit of detection, we re-analyzed our data changing all of our positive detections at ≤ 100 PPM to “not detected”. Simulating a less sensitive MRD assay in this way, ~ 17% (3/18) of patients that were classified as molecular progressors (specifically patients 503, 119, and 132) would no longer have observable molecular progression. For patient 106, in whom molecular progression was detected 65 days ahead of imaging, molecular progression would instead be detected at the same time as imaging, reducing the lead time to zero days. Finally, with the simulated limit of detection of 100 PPM, the lack of ctDNA clearance results in reduced association with PFS (HR: 6.76, 95% 2.32-19.4, p = 0.0004) and OS (HR: 3.11, 95% CI 1.14-8.53, p = 0.027) (Supplement Fig. 3).

## Discussion

The phase II KeyLargo study was initiated to examine the safety and efficacy of the PD-1 inhibitor pembrolizumab combined with oxaliplatin and capecitabine in patients with previously untreated HER2-negative metastatic esophagogastric adenocarcinoma^16^. Since then, several phase III trials have demonstrated clinical benefit from the addition of a PD-1 inhibitor to chemotherapy as first-line therapy for metastatic esophagogastric cancer with high PD-L1 combined positive score^3,17,18^. Nonetheless, even with selection by PD-L1 expression, a sizable proportion of patients do not respond to the combination of chemotherapy and PD-1 inhibitor, highlighting the need for better predictive biomarkers. In the KeyLargo study, the majority of patients achieved partial or complete responses, several of which were durable, but most patients eventually progressed, while a significant minority of patients did not respond to therapy at all^16^. Patients in the study were closely monitored with imaging assessments (RECIST 1.1, every three cycles). In addition, plasma was collected from patients at baseline, at several early time points, every three cycles, and around the time of clinical or radiographic progression. This rich biomarker sampling in a cohort of patients exhibiting a wide range of responses to therapy provided an opportunity to evaluate longitudinal relationships between dynamic plasma biomarkers and clinical outcomes. Leveraging these longitudinal plasma collections, we applied a novel, ultra-sensitive, tumor-informed ctDNA assay and explored its utility as a biomarker for early prediction of treatment response and early detection of disease progression.

In prior studies in early-stage EGC, ctDNA positivity after definitive chemoradiation or surgical resection was shown to associate with an increased risk of recurrence, shorter recurrence-free survival, and shorter disease-specific survival^11,19–22^. In the context of advanced EGC, baseline ctDNA levels correlate with the overall burden of disease^23,24^. In agreement with the literature, we found that 100% of the patients in the KeyLargo study had detectable ctDNA at baseline and all early time points up to C2D1, suggesting that patients are typically not completely clear of disease during this period, even in patients who eventually had durable CR/PR. In addition, ctDNA changes within the first 30 days of treatment predicted response to therapy. As early as one week after the initiation of pembrolizumab monotherapy (C1D8), patients who eventually responded to therapy (CR/PR) had a quantitatively larger (but not statistically significant) increase in ctDNA levels, potentially reflecting an increase in ctDNA due to tumor cell death. This suggests a possible association between the very short-term kinetics of ctDNA and subsequent therapeutic response that could be further explored in future studies. Importantly, from the initiation of therapy to the beginning of the second cycle (C2D1), patients who eventually responded to therapy (CR/PR) had a significantly greater decrease in ctDNA levels compared to patients who did not respond to therapy (SD/PD), suggesting that ctDNA dynamics at this early time point can predict response to therapy before the first imaging assessment. By longitudinally correlating changes in plasma ctDNA levels and tumor burden on imaging, we further demonstrated that ctDNA levels correlate with tumor burden in patients with advanced EGC not only at baseline but also throughout the treatment course. There are also cases where the ctDNA levels may be a more accurate reflection of patient disease status than imaging, as exemplified by patient 108 (Fig. 6D). Together, our results support the use of ctDNA as a non-invasive biomarker of treatment response and suggest that ctDNA and imaging may provide complementary information in advanced EGC.

We also examined the association of ctDNA dynamics with survival and the utility of ctDNA for the early detection of progression. Prior studies in advanced EGC have shown that high baseline ctDNA levels are associated with inferior survival^23,25^ and that ctDNA dynamics during systemic therapy with chemotherapy or immunotherapy are associated with prognosis^24,26–28^. In our study, we expanded on these studies with a more granular understanding of ctDNA dynamics and their correlation with clinical outcomes starting as early as 30 days (C2D1) after the initiation of immunotherapy in metastatic EGC. We found that both molecular response (50% or greater decrease in ctDNA levels by C4D1) and ctDNA clearance (undetectable ctDNA at any time through C7D1) were associated with improved PFS and OS. In addition, a 20% or greater increase in ctDNA levels served as an early biomarker of molecular progression, with a median lead time of 65 days relative to radiographic progression. This extended lead time is of significant clinical importance, as it offers a valuable window for potential early intervention and timely treatment adjustments for advanced EGC patients. Finally, we noted the impact of an ultra-sensitive MRD assay with a limit of detection as low as 1 to 3 PPM of ctDNA, which is significantly more analytically sensitive compared to other commercially available MRD tests. Even in metastatic EGC patients who can have high ctDNA shedding levels, ~ 20% of the positive ctDNA detections in this study were in the ultra-low range of 1 to 100 PPM. A significant number of these detections would likely have been missed by a less sensitive MRD assay. Our simulated analysis showed that use of a less sensitive MRD assay could have led to a reduced association between ctDNA clearance and survival, decreased lead time, and missed molecular progression events. This analysis demonstrates the importance of an ultra-sensitive MRD assay for tracking ctDNA dynamics consistently, classifying patient responses accurately, and detecting progression early.

In summary, we used an ultra-sensitive, tumor-informed ctDNA assay to investigate the role of ctDNA as a biomarker of survival, response, and progression in patients with metastatic HER2-negative esophagogastric adenocarcinoma treated with pembrolizumab and chemotherapy in the KeyLargo study. Early changes in ctDNA levels differed in patients who responded to therapy versus non-responders. Furthermore, by examining longitudinal plasma ctDNA dynamics on treatment, we found that changes in ctDNA levels served as an early biomarker of disease progression and were prognostic of survival. Finally, this study shows that even in the setting of advanced EGC, it is critically important to utilize a ctDNA MRD test with sensitivity down to the 1 PPM range to accurately track and predict response to therapy. Our findings suggest that serial plasma ctDNA monitoring with an ultra-sensitive MRD test in patients with advanced EGC may have clinical utility as a non-invasive biomarker to assess prognosis, predict treatment response, and detect disease progression.

## Methods

### KeyLargo trial

The initial KeyLargo cohort comprised 36 advanced EGC patients who were administered a first-line treatment regimen combining oxaliplatin, capecitabine, and pembrolizumab^16^. After an initial 7-day monotherapy regimen of pembrolizumab, the treatment cycles adhered to a standard 21-day schedule of oxaliplatin on day 1 and capecitabine on days 1–14. After the initial dose on C1D1, pembrolizumab was given on day 1 of each 21-day cycle starting with cycle 2. Plasma samples were collected on C1D8 (after pembrolizumab monotherapy), at the end of cycle 1, and at each restaging cycle, aligning with regularly scheduled imaging and RECIST evaluations (Fig. 1). This clinical study was approved by the Institutional Review Board and conducted in accordance with the Declaration of Helsinki guidelines.

### Patients

A total of 33 EGC patients in KeyLargo were evaluable for RECIST best response. Notably, 6/33 (18%) of patients achieved a complete response (CR), 19/33 (58%) exhibited a partial response (PR), 3/33 (9%) exhibited stable disease (SD), and 5/33 (15%) exhibited progressive disease (PD). The median overall survival (OS) was 488 days, and the median progression-free survival (PFS) was 230 days. All patients provided written informed consent regarding sample collection and further analysis.

#### Sample collection and processing

Formalin-fixed, paraffin-embedded (FFPE) tumor specimens were collected at baseline from 25 patients in KeyLargo. Peripheral blood samples were collected from consenting patients at baseline, at 7 days after pembrolizumab monotherapy, at the end of the first cycle, and at each restaging. Blood was collected into EDTA vacutainers, processed on-site within 2 hours, and platelet poor EDTA plasma was isolated using standard processing procedures^29^. Plasma was aliquoted, frozen, and stored at −80°C until use. All sample and data handling procedures adhered to Guidelines for Good Clinical Practice (GCP) and institutional standard operating procedures (SOPs).

### Tumor-informed, bespoke ctDNA profiling for MRD

Out of the 36 KeyLargo patients, 25 patients were analyzed for presence and quantification of ctDNA in plasma. Reasons for exclusion included absence of FFPE blocks (n = 4), poor DNA yields (n = 5), and low tumor content (n = 2) (Fig. 1). Sample preparation and characterization for NeXT Personal® was performed as previously described^12^. In brief, tumor and normal tissue samples were collected and processed following standard procedures. Whole-genome sequencing (WGS) from tumor and normal samples was performed at approximately 30x to design hybrid capture probe panels with up to 1,800 somatic MRD variants. Cell-free DNA libraries were enriched with patient-specific MRD panels, and the ctDNA detection status for each plasma sample was determined using a one-tailed Poisson test. Samples were classified as ctDNA-positive (i.e., “detected”) if the tumor signal was significantly (*p* ≤ 0.001) above the expected noise. Several patients were excluded from post-WGS analysis due to limited tumor content below 7% (Supplement Fig. 2), as estimated from tumor-normal WGS data.

### Statistics

Panel design and all ctDNA measures were completed while blinded to clinical variables, RECIST determinations, and patient survival, until final analysis. Statistical analyses were performed using R (version 4.3.1; R Foundation for Statistical Computing, Vienna, Austria).

Changes at the time points of interest were analyzed and compared to baseline measurements unless otherwise specified. Samples in which the best overall response was SD/PD were grouped and compared against patients displaying CR/PR. The Wilcoxon rank-sum test was used to compare the change in ctDNA levels from baseline to C1D8 or C2D1 in the SD/PD versus CR/PR groups. Additional cycles were not assessed due to the small representation of SD/PD samples.

The cohort was divided into subgroups based on the observed ctDNA dynamics, distinguishing between patients with a significant decrease in ctDNA levels and those with persistent or increasing levels. Kaplan-Meier curves were constructed to visually depict the PFS and OS probabilities over time for distinct subgroups categorized by ctDNA dynamics. The log-rank test was applied to assess the statistical significance of differences in survival distributions among the ctDNA dynamic subgroups. The Cox proportional hazards regression model was used to test the association between ctDNA subgroups and PFS/OS.

To investigate the role of ctDNA as a prognostic biomarker associated with PFS and OS, patients were grouped according to two different criteria. I) First, patients were grouped based on the change in ctDNA levels from baseline to C2D1 or C4D1. If ctDNA levels were available at both C2D1 and C4D1, the earlier time point (C2D1) was used. Patients with a greater than 50% decrease in ctDNA level at this time point relative to baseline were defined as having a “molecular response” (mR). Patients with an increase, no change, or a less than 50% decrease in ctDNA level at this time point relative to baseline were defined as having “molecular non-response” (mNR). II) Alternatively, patients were grouped according to whether they had ctDNA clearance, defined as the absence of ctDNA signal at any time point between C1D8 and C7D1. Since all patients had detectable ctDNA at C1D8 and C2D1, ctDNA clearance was effectively assessed between C4D1 and C7D1. Thus, patients displaying a ctDNA signal below the limit of detection at any of those time points were categorized as having “ctDNA clearance.”

Lead time was determined as the time difference, in days, between imaging progression and molecular progression. Negative values represent earlier detection of progression by ctDNA. Imaging progression was defined as the earliest detection of PD by RECIST 1.1 criteria for each patient. Molecular progression was defined as the earliest ctDNA increase of 20% or more from the lowest ctDNA measurement. Patients with gaps of larger than 30 days between imaging and ctDNA measurements were excluded from the lead time analysis. In addition, lead time could not be calculated for patients who did not experience imaging or molecular progression (the 2 patients who achieved CR) or who experienced imaging progression but had no additional plasma time points post-progression to capture molecular progression (patient 140).

Similarly, when correlating ctDNA dynamics with imaging dynamics, only time points containing both measurements within 30 days were kept. The delta in imaging-based tumor size was calculated as the ratio of the sum of the largest diameters of all lesions at the time point of interest over the baseline sum of the largest diameters. Similarly, ctDNA ratio is defined as the ratio of ctDNA PPM at the time point of interest and ctDNA PPM at baseline. The correlation between the changes in ctDNA and imaging-based tumor size was tested using Spearman’s rank correlation.

## Supplementary Material

Supplement 1

## Data Availability

The datasets generated and analyzed during the current study are available in the Open Science Framework (OSF) repository at https://osf.io/uqjbm/.

## References

[1] Bray F. Global cancer statistics 2022: GLOBOCAN estimates of incidence and mortality worldwide for 36 cancers in 185 countries. CA Cancer J Clin 74, 229–263, doi:10.3322/caac.21834 (2024). 38572751

[2] Van Cutsem E., Sagaert X., Topal B., Haustermans K. & Prenen H. Gastric cancer. Lancet 388, 2654–2664, doi:10.1016/S0140-6736(16)30354-3 (2016). 27156933

[3] Janjigian Y. Y. First-line nivolumab plus chemotherapy versus chemotherapy alone for advanced gastric, gastro-oesophageal junction, and oesophageal adenocarcinoma (CheckMate 649): a randomised, open-label, phase 3 trial. Lancet 398, 27–40, doi:10.1016/S0140-6736(21)00797-2 (2021). 34102137 PMC8436782

[4] Chau I. Nivolumab (NIVO) plus ipilimumab (IPI) or NIVO plus chemotherapy (chemo) versus chemo as first-line (1L) treatment for advanced esophageal squamous cell carcinoma (ESCC): First results of the CheckMate 648 study. J Clin Oncol 39, 2021 (suppl 15; abstr LBA4001), doi:10.1200/JCO.2021.39.15_suppl.LBA4001 (2021).

[5] Genovese G. Clonal hematopoiesis and blood-cancer risk inferred from blood DNA sequence. N Engl J Med 371, 2477–2487, doi:10.1056/NEJMoa1409405 (2014). 25426838 PMC4290021

[6] Huang R. S. P. Circulating Cell-Free DNA Yield and Circulating-Tumor DNA Quantity from Liquid Biopsies of 12 139 Cancer Patients. Clin Chem 67, 1554–1566, doi:10.1093/clinchem/hvab176 (2021). 34626187

[7] Einbinder Y. Elevated Circulating Cell-Free DNA in Hemodialysis-Treated Patients Is Associated with Increased Mortality. Am J Nephrol 51, 852–860, doi:10.1159/000510771 (2020). 33105130

[8] Nadauld L. D. The PATHFINDER Study: Assessment of the Implementation of an Investigational Multi-Cancer Early Detection Test into Clinical Practice. Cancers (Basel) 13, doi:10.3390/cancers13143501 (2021). PMC8304888 34298717

[9] Alese O. B. Circulating Tumor DNA: An Emerging Tool in Gastrointestinal Cancers. Am Soc Clin Oncol Educ Book 42, 1–20, doi:10.1200/EDBK_349143 (2022). 35471832

[10] Yang J. Deep sequencing of circulating tumor DNA detects molecular residual disease and predicts recurrence in gastric cancer. Cell Death Dis 11, 346, doi:10.1038/s41419-020-2531-z (2020). 32393783 PMC7214415

[11] Huffman B. M. Analysis of Circulating Tumor DNA to Predict Risk of Recurrence in Patients With Esophageal and Gastric Cancers. JCO Precis Oncol 6, e2200420, doi:10.1200/PO.22.00420 (2022). 36480779 PMC10530958

[12] Northcott J. Analytical validation of NeXT Personal(R), an ultra-sensitive personalized circulating tumor DNA assay. Oncotarget 15, 200–218, doi:10.18632/oncotarget.28565 (2024). 38484152 PMC10939476

[13] Ryoo S. B. Personalised circulating tumour DNA assay with large-scale mutation coverage for sensitive minimal residual disease detection in colorectal cancer. Br J Cancer 129, 374–381, doi:10.1038/s41416-023-02300-3 (2023). 37280413 PMC10338477

[14] O’Sullivan H. M., Feber A. & Popat S. Minimal Residual Disease Monitoring in Radically Treated Non-Small Cell Lung Cancer: Challenges and Future Directions. Onco Targets Ther 16, 249–259, doi:10.2147/OTT.S322242 (2023). 37056631 PMC10089274

[15] Abbosh C. Tracking early lung cancer metastatic dissemination in TRACERx using ctDNA. Nature 616, 553–562, doi:10.1038/s41586-023-05776-4 (2023). 37055640 PMC7614605

[16] Uronis H. E. (American Society of Clinical Oncology, 2021).

[17] Sun J. M. Pembrolizumab plus chemotherapy versus chemotherapy alone for first-line treatment of advanced oesophageal cancer (KEYNOTE-590): a randomised, placebo-controlled, phase 3 study. Lancet 398, 759–771, doi:10.1016/S0140-6736(21)01234-4 (2021). 34454674

[18] Rha S. Y. Pembrolizumab plus chemotherapy versus placebo plus chemotherapy for HER2-negative advanced gastric cancer (KEYNOTE-859): a multicentre, randomised, double-blind, phase 3 trial. Lancet Oncol 24, 1181–1195, doi:10.1016/S1470-2045(23)00515-6 (2023). 37875143

[19] Leal A. White blood cell and cell-free DNA analyses for detection of residual disease in gastric cancer. Nat Commun 11, 525, doi:10.1038/s41467-020-14310-3 (2020). 31988276 PMC6985115

[20] Kim Y. W. Monitoring circulating tumor DNA by analyzing personalized cancer-specific rearrangements to detect recurrence in gastric cancer. Exp Mol Med 51, 1–10, doi:10.1038/s12276-019-0292-5 (2019). PMC6802636 31395853

[21] Azad T. D. Circulating Tumor DNA Analysis for Detection of Minimal Residual Disease After Chemoradiotherapy for Localized Esophageal Cancer. Gastroenterology 158, 494–505 e496, doi:10.1053/j.gastro.2019.10.039 (2020). 31711920 PMC7010551

[22] Ococks E. Serial Circulating Tumor DNA Detection Using a Personalized, Tumor-Informed Assay in Esophageal Adenocarcinoma Patients Following Resection. Gastroenterology 161, 1705–1708 e1702, doi:10.1053/j.gastro.2021.07.011 (2021). 34284036 PMC8586817

[23] Fang W. L. Clinical significance of circulating plasma DNA in gastric cancer. Int J Cancer 138, 2974–2983, doi:10.1002/ijc.30018 (2016). 26815009

[24] van Velzen M. J. M. Circulating tumor DNA predicts outcome in metastatic gastroesophageal cancer. Gastric Cancer 25, 906–915, doi:10.1007/s10120-022-01313-w (2022). 35763187 PMC9365750

[25] Maron S. B. Circulating Tumor DNA Sequencing Analysis of Gastroesophageal Adenocarcinoma. Clin Cancer Res 25, 7098–7112, doi:10.1158/1078-0432.CCR-19-1704 (2019). 31427281 PMC6891164

[26] Normando S. R. C. Circulating free plasma tumor DNA in patients with advanced gastric cancer receiving systemic chemotherapy. BMC Clin Pathol 18, 12, doi:10.1186/s12907-018-0079-y (2018). 30498396 PMC6258437

[27] Bratman S. V. Personalized circulating tumor DNA analysis as a predictive biomarker in solid tumor patients treated with pembrolizumab. Nat Cancer 1, 873–881, doi:10.1038/s43018-020-0096-5 (2020). 35121950

[28] Kim S. T. Comprehensive molecular characterization of clinical responses to PD-1 inhibition in metastatic gastric cancer. Nat Med 24, 1449–1458, doi:10.1038/s41591-018-0101-z (2018). 30013197

[29] Nixon A. B. Predictive Biomarkers of Overall Survival in Patients with Metastatic Renal Cell Carcinoma Treated with IFNalpha +/− Bevacizumab: Results from CALGB 90206 (Alliance). Clin Cancer Res 28, 2771–2778, doi:10.1158/1078-0432.CCR-21-2386 (2022). 34965953 PMC9240110

