# Supplementary material for: Ultra-sensitive, tumor-informed ctDNA profiling in pembrolizumab-treated esophagogastric cancer patients predicts clinical responses
Source: Res Sq. 2024 Dec 18:rs.3.rs-5349536. Preprint. [Version 1] doi: 10.21203/rs.3.rs-5349536/v1 (PMC11702795; doi:10.21203/rs.3.rs-5349536/v1)
Supplement: Supplement 1 [file NIHPPRS5349536v1-supplement-1.pdf]

Supplementary Files

This is a list of supplementary files associated with this preprint. Click to download.

- SupplementalFiguresandTablesFinal.docx
